# Guidelines and considerations for the use of system suitability and quality control samples in mass spectrometry assays applied in untargeted clinical metabolomic studies

**DOI:** 10.1007/s11306-018-1367-3

**Published:** 2018-05-18

**Authors:** David Broadhurst, Royston Goodacre, Stacey N. Reinke, Julia Kuligowski, Ian D. Wilson, Matthew R. Lewis, Warwick B. Dunn

**Affiliations:** 1 School of Science, Centre for Integrative Metabolomics and Computational Biology, Edith Cowan University, Joondalup, Perth, Australia; 2 School of Chemistry, Manchester Institute of Biotechnology, University of Manchester, Manchester, M1 7DN UK; 3 Separation Sciences and Metabolomics Laboratory, Murdoch University, Perth, WA Australia; 4 Neonatal Research Unit, Health Research Institute La Fe, Avda. Fernando Abril Martorell 106, 46026 Valencia, Spain; 5 Division of Computational and Systems Medicine, Department of Surgery and Cancer, Imperial College London, Sir Alexander Fleming Building, Exhibition Road, South Kensington, London, SW7 2AZ UK; 6 School of Biosciences, University of Birmingham, Edgbaston, Birmingham, B15 2TT UK; 7 Phenome Centre Birmingham, University of Birmingham, Edgbaston, Birmingham, B15 2TT UK; 8 Institute of Metabolism and Systems Research, University of Birmingham, Edgbaston, Birmingham, B15 2TT UK

**Keywords:** Quality assurance (QA), Quality control (QC), System suitability samples, Pooled QC samples, Standard reference materials (SRMs), Long-term reference (LTR) QC samples

## Abstract

**Background:**

Quality assurance (QA) and quality control (QC) are two quality management processes that are integral to the success of metabolomics including their application for the acquisition of high quality data in any high-throughput analytical chemistry laboratory. QA defines all the planned and systematic activities implemented before samples are collected, to provide confidence that a subsequent analytical process will fulfil predetermined requirements for quality. QC can be defined as the operational techniques and activities used to measure and report these quality requirements after data acquisition.

**Aim of review:**

This tutorial review will guide the reader through the use of system suitability and QC samples, why these samples should be applied and how the quality of data can be reported.

**Key scientific concepts of review:**

System suitability samples are applied to assess the operation and lack of contamination of the analytical platform prior to sample analysis. Isotopically-labelled internal standards are applied to assess system stability for each sample analysed. Pooled QC samples are applied to condition the analytical platform, perform intra-study reproducibility measurements (QC) and to correct mathematically for systematic errors. Standard reference materials and long-term reference QC samples are applied for inter-study and inter-laboratory assessment of data.

## Introduction

Clinical metabolomics (otherwise known as metabonomics or metabolic phenotyping) is a rapidly growing field of research, primarily focused on the investigation of human health (Dunn et al. 2015), disease (Xie et al. 2014) and ageing (Menni et al. 2013), with diverse clinical application in areas such as prognostic biomarkers (Rhee et al. 2016; Shah et al. 2012; O’Gorman and Brennan 2017), pathophysiological mechanisms (Kirpich et al. 2016; Terunuma et al. 2014; Drenos et al. 2016), and stratified medicine (Kaddurah-Daouk and Weinshilboum 2015). Depending on the research question, or specific application, there are three commonly used analytical metabolomics strategies (Dunn et al. 2011a):

- *Untargeted assays* where the objective is to reproducibly measure as many metabolites as is feasible [typically low thousands of metabolites when (multiple) MS platforms are applied], provide semi-quantitative data (chromatographic peak areas are reported, not concentrations), and where the chemical identity of metabolites is not necessarily known before data are acquired (post hoc identification is performed applying full-scan and MS/MS data acquired during the assays).
- *Targeted assays* where the focus is on a small number of biologically important metabolites whose chemical identity is known prior to data acquisition, and for which an absolute concentration of each metabolite is reported through the use of isotopically-labelled internal standards (calibration curves constructed with authentic chemical standards and isotopically-labelled internal standards for each targeted metabolite) or via the standard addition method.
- *Semi-targeted assays* which act as an intermediate between untargeted and targeted methodologies, where low hundreds of metabolites are targeted, whose chemical identity is known prior to data acquisition, and for which semi-quantification is applied to define approximate metabolite concentrations (typically applying one calibration curve and internal standard for multiple metabolites).

Quality assurance (QA) and quality control (QC) are two quality management processes that are integral to the success of any research study, and in the context of metabolomics, they are critical for the acquisition of high quality data in any high-throughput analytical chemistry laboratory. According to ISO9000 (ISO9000 2015), QA addresses the activities the laboratory undertakes to provide confidence that quality requirements will be fulfilled, whereas QC describes the individual measures which are used to actually fulfil the requirements. These definitions have been endorsed by CITAC (the Cooperation on International Traceability in Analytical Chemistry) and EuraChem (A Focus for Analytical Chemistry in Europe) (Barwick 2016).

QA can also be defined, from a more chronological perspective, as all the planned and systematic activities implemented *before* samples are collected, to provide confidence that a subsequent analytical process will fulfil predetermined requirements for quality. Such activities will include: formal design of experiment (DoE); certified and documented staff training; standard operating procedures for biobanking, sample handling, and instrument operation; preventative instrument maintenance; and standardised computational workflows. Correspondingly, QC can be defined as the operational techniques and activities used to measure and report these quality requirements *during and after* data acquisition.

Laboratories running targeted and semi-targeted metabolomics assays may adopt established guidelines defining both the expected quality of data and the processes to measure and report this quality. The most commonly applied guidelines are those published by the Food and Drug Administration titled: “Guidance for Industry: Bioanalytical Method Validation” (FDA 2001). Whilst these guidelines were originally developed for targeted drug analysis, the general principles they encompass can be adapted, with care, to multi-analyte targeted (and semi-targeted assays). More recently, further guidance has been developed for the measurement of biomarkers (usually proteins) with slightly different acceptance criteria (Lowes and Ackermann 2016).

Whilst these guidelines provide a good practical foundation for metabolomics system suitability and QA/QC processes they were not designed with metabolomics in mind, and although readily adaptable to (semi-) targeted methods, they are not easily translated into a form usable for untargeted metabolomics. As such, currently there are no community agreed-upon guidelines for QA, and there is very little consistency in the system suitability and QC methods for assessing system performance and reporting data quality. A recent review article has comprehensively discussed QA processes in untargeted metabolomics (Dudzik et al. 2018).

In targeted and semi-targeted assays, the use of QC samples for assessing data quality is common practice (FDA 2001). Similar approaches can be applied for untargeted assays. In 2006, the introduction of a pragmatic approach to the use of pooled QC samples for within-study reporting of data quality helped drive the QC processes forward in this area (Sangster et al. 2006). This initial work was further developed with recommendations as to how the data from such QCs could be analysed, (Gika et al. 2007) and numerous papers and reviews have emerged from this early introduction (for example see, Dunn et al. 2011b, 2012; Godzien et al. 2015). The importance of QA and QC in the metabolomics community is further illustrated through the establishment of the Data Quality task group of the International Metabolomics Society (Bearden et al. 2014; Dunn et al. 2017), and the convening of a NCI-funded *Think Tank on Quality Assurance and Quality Control in Untargeted Metabolomics Studies* in 2017. A recent questionnaire on training in metabolomics also highlighted the unmet need for training in QA and QC processes (Weber et al. 2015).

In this paper, we will focus on the two areas of system suitability and quality control. That is, the types of samples applied to untargeted metabolomics workflows in order to demonstrate system suitability prior to data acquisition and QC samples applied to demonstrate analytical accuracy, precision, and repeatability after data processing and which can be converted to metrics describing data quality. We will describe complementary types of system suitability and QC sample, each having its own specific utility, but when combined can provide a robust and effective analytical system suitability and QC protocol. We will discuss the motivation for each sample type, followed by recommendations on how to prepare the samples, how the resulting data are assessed to report quality, and how these samples can be integrated in to single or multi-batch analytical experiments.

## Types of system suitability and quality control tasks

### System suitability testing

In order to yield specimens of high intrinsic value, the collection of biological samples in a clinical study requires careful planning, recruitment, financial support, and investment of time. As such, it is imperative that actions are put in place to minimise the loss of potentially irreplaceable biological samples as they pass through the metabolomics analytical pipeline, from sample preparation to data processing. Therefore, prior to the analysis of any biological sample, the suitability of a given analytical platform for imminent sample analysis should be assessed, and thus its analytical performance assured. This initial process can be accomplished by performing a set of activities involving system suitability samples and blank samples designed to test analytical performance metrics that will qualify the instrument as “fit for purpose” before biological test samples are analysed. The simplest approach for system suitability checks are to first run a “blank” gradient with no sample as this will reveal problems due to impurities in the solvents or contamination of the separations system including the LC/GC/CE column.

**Fig. 1:**
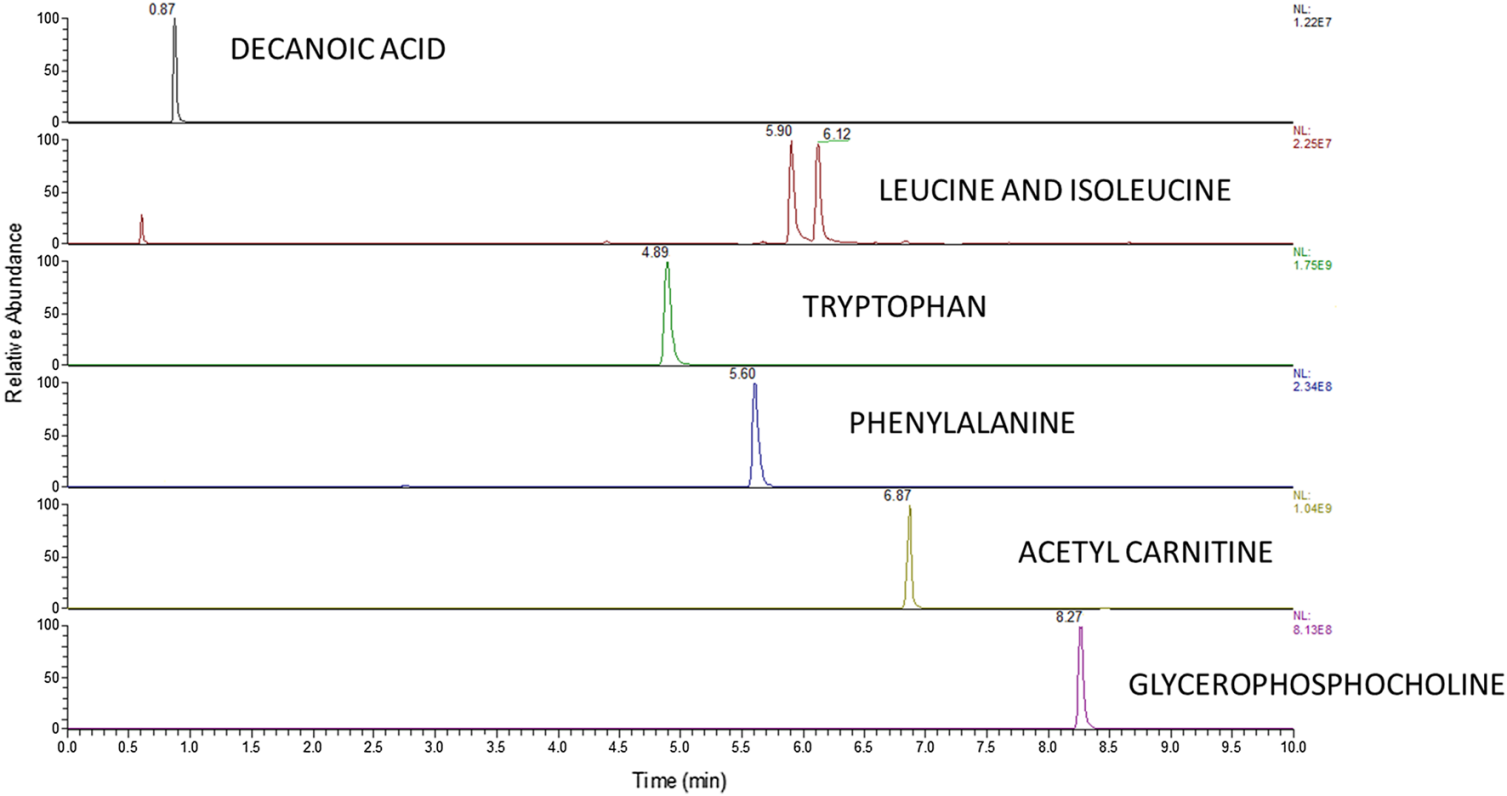
Example of typical data acquired for a system suitability sample. Here, a seven component system suitability sample has been applied in a HILIC positive ion assay and includes an early elution metabolite (decanoic acid) and later elution metabolites. Leucine and isoleucine are included to assess chromatographic resolving power for isomers. The base peak chromatograms are shown for each metabolite to assess peak symmetry with retention time and *m*/*z* calculated to assess chromatographic stability and mass accuracy

If clean, the analysis of a solution containing a small number of authentic chemical standards (typically five to ten analytes) dissolved in a chromatographically suitable diluent, from which the acquired data can be quickly assessed for accuracy and precision in an automated computational approach (for example, Dunn et al. 2011b). Importantly, as these analytes are not in a biological matrix, they act to assess the instrument as a clean sample devoid of biological matrix effects. The most appropriate solution will contain analytes which are distributed as fully as possible across the *m*/*z* range and the retention time range so to assess the full analysis window. The results for this sample are assessed for the mass-to-charge (*m*/*z*) ratio and chromatographic characteristics, including retention time, peak area, and peak shape (e.g. tailing factor) and compared to pre-defined acceptance criteria. In cases where the acceptance criteria are fulfilled then sample processing and data acquisition can be initiated. In cases where the acceptance criteria are not fulfilled then corrective maintenance of the analytical platform should be performed and the system suitability check solution reanalysed. An example of acceptance criteria to apply are: (i) *m*/*z* error of 5 ppm compared to theoretical mass, (ii) retention time error of < 2% compared to the defined retention time, (iii) peak area equal to a predefined acceptable peak area ± 10% and (iv) symmetrical peak shape with no evidence of peak splitting. Acceptance criteria can be tailored to laboratory specific requirements for each analytical assay and no community-agreed acceptance criteria for untargeted metabolomics are currently reported. As a secondary check, a system suitability sample can be analysed at the end of each batch to act as a rapid indicator of intermediate system level quality failure, before proceeding to time-consuming and in-depth data analysis. Figure 1 shows a base peak chromatogram for a seven-component system suitability sample analysed using a HILIC UHPLC-MS platform.

### System suitability blank and process blank samples

Untargeted metabolomics, by definition, attempts to be unbiased; although we note the metabolite extraction method and chosen analytical platform used will influence the types of small molecules enriched and detected based on their physicochemical properties. Therefore, the associated analytical methodologies aim to maximise the number, and physicochemical diversity, of metabolites detected in a biological sample. An undesirable, but unavoidable, by-product of this comprehensive “catch all” approach is that the resulting data may unintentionally include signals from chemicals present in mobile phases, together with contaminants derived from sample collection, sample handling, and sample processing consumables.

To ensure that the data matrix used for statistical analysis and biological interpretation accurately reflects the biological system being studied, signals derived from these sources need to be identified and then removed, constrained, or labelled. This is particularly the case for the analysis of volatile metabolites (e.g. from breath) as plastics used during collection and column bleed from siloxanes is hard to negate. To achieve this a “blank” sample preparation process can be performed applying the same solvents, chemicals, consumables, and standard operating procedure, as for the test samples, but in the absence of any actual biological sample. These “blank” samples are commonly known as *process blanks*, or *extraction blanks*. This is a third type of system suitability sample when analysed at the start of an analytical batch to assess the suitability of the system. Sample processing can involve dilution (e.g. urine), biochemical precipitation (e.g. applying organic solvents to precipitate proteins, RNA and DNA in plasma) and extraction (e.g. tissue homogenisation and metabolite extraction in to an extraction solution). Any detected signals in these blank processed samples can be confidently identified as contaminants and dealt with appropriately as described below.

Another undesirable artefact, known as “carryover”, needs to be tested for after each analytical run. Here, signals from one biological test sample are also detected in (carried over to) the next biological test sample. For example, this is usually the result of inadequate washing of the LC injection system between sample injections. This source of contamination can be investigated by injecting a blank sample after a series of test samples, and then look for the appearance of sample-related signals in that blank sample. Unlike above where a blank sample is analysed at the start of the run to assess system suitability, this blank sample is a QC sample to assess influence of blank signal on data quality. Any sample-based signal in the blank extraction samples can be confidently identified as carryover contaminant and dealt with appropriately.

After deconvolution of all raw spectra into a data matrix, peaks observed in the blank samples satisfying a specific exclusion criterion may require the deletion of the associated peak from the data matrix. Theoretical exclusion criteria are listed below but no defined criteria have been reported or agreed as a standard to apply in the metabolomics community and the authors only recommend that the criteria used are reported. These theoretical exclusion criteria could be (a) the signal in the blank sample is great than a predefined threshold (e.g. above 10× the expected background noise signal), (b) the signal in the blank sample is greater than a percentage of the average signal from the complete set of biological samples. (e.g. a 5% of median acceptance criteria could be set, thus any peaks with a “blank” signal > 5% of the median are removed from the dataset), or (c) calculate the blank contribution but do not remove the peak prior to data analysis; instead, the peak is flagged as “potentially contaminated”. If the peak is subsequently defined as biologically important, a balanced decision as to whether the blank-related contribution influences the impact/quality of the associated peak can be made. As no definitive recommendation is currently agreed upon, the acceptance criteria used in a study should be reported in publications, and public data repositories.

### Pooled QC sample(s) for intra-study assessment of data

A number of published reports have discussed the use of pooled QC samples and we will discuss in greater depth below (Dudzik et al. 2018; Sangster et al. 2006; Gika et al. 2007; Dunn et al. 2011b, 2012; Godzien et al. 2015; Lewis et al. 2016). From an analytical chemistry perspective, untargeted metabolomics has two seemingly paradoxical aspirations. Methodologies aim to maximise both the number and diversity of measured metabolites across several orders of concentration magnitude, whilst simultaneously generating high precision, repeatable, and reproducible data. This dichotomy is particularly challenging, given that untargeted methodologies are “blind”, and all of the acquired data are simply “peaks” until they are aligned/grouped/filtered and ultimately identified in an extensive and mathematically complex computational pipeline.

With untargeted metabolomics, where hundreds or thousands of metabolites are detected, and the chemical identity of all metabolites is not known prior to data acquisition, it is impossible to use internal standards comprehensively and it is also impossible to generate metabolite specific calibration curves. Consequentially, it is impossible to provide absolute quantification, absolute estimates of accuracy (how close the measured concentration is to the real concentration), absolute estimates of precision (random error in quantification over repeated measurement of an identical biological specimen), with no clearly defined limits of detection, limits of quantification, and linearity quantifiers.

A high quality, multi-analyte targeted assay may take months to develop. However, all the effort invested in initial development, is returned by way of clear QA acceptance criteria, and relatively simple QC protocols (FDA 2001). QC samples can simply consist of a mixture of the authentic chemical standard representing the target analytes and associated isotopically labelled internal standards, of a fixed concentration, spiked into the test sample matrix, where each QC sample is, to a very high degree of confidence, identical. Then, acquiring quantitative measurement of all the targeted metabolites for a small number of QC samples, distributed evenly in an analytical batch, can quickly generate the required acceptance criteria metrics. This process may be repeated for different concentrations of analyte (typically as QC-low, QC-medium, QC-high). After an analytical batch, if the calculated QC measures are within the predefined tolerances (acceptance criteria) for precision and accuracy, and the acquired data for the test samples are within the linear calibration range, then the data are deemed fit for purpose and statistical analysis can begin, otherwise the assay failed, and system diagnostic tests need to be performed before reanalysing (and reprocessing) the biological samples.

Providing similar tangible metrics for ensuring that an untargeted metabolomics assay is performing effectively is much more difficult. There are no predetermined QC acceptance criteria for each detected metabolite, and no limits of quantification. In fact, the only solution is to shift a large proportion of the effort traditionally directed toward generating quality assurance processes (for example, internal standards and calibration curves), over to providing more comprehensive quality control protocols and introduce the concept of cleaning data. Unfortunately, this is not straightforward as QC for untargeted metabolite quantification is severely constrained. Of all the metrics of quality mentioned thus far, for untargeted assays, it is **only** possible to provide a relative measure of precision—*the random error in quantification over repeated measurement of a biologically identical sample*. The choice of “biologically identical sample” (QC sample) is also limited. The composition of the QC sample should reflect the aggregate metabolite composition of all of the biological samples in a given study. The sample matrix composition is also important because this can provide variability in the response measured through its interaction with the analytical platform (e.g. through matrix-specific and sample-specific ionisation suppression). It is important to note that if a metabolite is not present in the pooled QC sample then the quality of its measurement cannot be calculated and reported.

The most appropriate way to create multiple copies of such a complex QC sample is to use the biological test samples themselves. One could simply sub-aliquot each test sample into replicates (e.g. *n* = 3), then randomise the order of processing and injection for the new sample set. After data acquisition and spectral deconvolution into a metabolite data matrix (*N* samples × *P* metabolite features), measures of repeatability can be calculated for each set of replicates, and then aggregated into a single measure of precision. This strategy however, comes with the considerable cost of greatly increased total analysis time as it triples the number of biological sample injections.

**Fig. 2:**
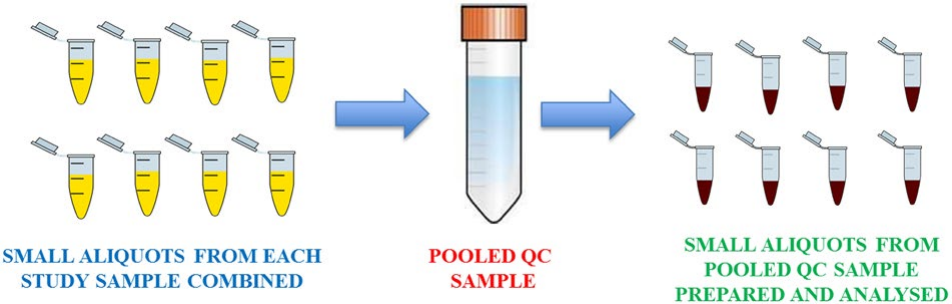
Visualisation of how a pooled QC sample is prepared from aliquots of the study biological samples from which aliquots of the pooled QC sample are extracted for analysis in an identical manner as for the study biological samples

An alternative and more time-efficient method of using the test samples themselves as the source for QC samples, is to generate multiple replicates of a single test sample (*n* > 5). These replicates would then be evenly distributed through the analytical batch. At the end of data processing, a single measure of precision can then be calculated for each metabolite feature. One clear problem with this method is the assumption that a single QC sample has a suitably representative metabolite composition, both in terms of number and concentration of metabolites and matrix species. A very simple work-around for this problem is to generate a single pooled QC sample from all, or a representative subset, of the biological test samples in a given study. This is achieved by taking a small volume of each biological test sample, thoroughly mixing into a homogenous pooled sample, and then preparing multiple aliquots from that pooled sample, thus generating a set of “pooled QC” samples (see Fig. 2). There are several variations on this basic premise of a pooled QC, and they are summarised in Table 1.

**Table 1:** A summary of different types of pooled QC samples and their associated advantages and limitations

Option	Type of pooled QC sample	Preparation method	Typical sample types	Comments
1	A pooled QC sample created from ALL of the biological test samples	A small aliquot of each biological test sample is pooled in to a single QC sample followed by sample processing of aliquots of the pooled sample in an identical approach as the biological test samples	Biofluids where adequate volumes are available including urine and plasma/serum	(1) Applied for biofluids where suitable sample volumes are available for all samples; (2) the most representative pooled QC sample for a biological study
2	A pooled QC sample created from a representative SUBSET of the biological test samples	A small aliquot of a representative subset of biological test samples are pooled in to a single QC sample followed by sample processing of aliquots of the pooled sample in an identical approach as the biological test samples	Biofluids where adequate volumes are available including urine and plasma/serum	(1) Applied for biofluids where suitable sample volumes are available for some samples; (2) applied when data acquisition of samples collected in beginning of project is started before all samples have been collected; (3) the most representative pooled QC sample for a biological study with the exception of option 1; (4) uses smaller sample volumes for a subset of samples; (5) can be applied to prepare a representative pooled QC sample for each biological class where the composition of different samples in different biological classes is very different
3	A pooled sample created from the same sample type but from a DIFFERENT BIOLOGICAL SOURCE	A small aliquot of each biological sample acquired from a different biological source are pooled in to a single QC sample followed by sample processing of aliquots of the pooled sample in an identical approach as the biological test samples	Any sample type	(1) Applied for biofluids where small sample volumes are available; (2) typically applied when insufficient sample volume available to prepare pooled QC sample; (3) the metabolites present, their concentration and the sample matrix is not an average of the biological test samples
4	A pooled sample created from the PROCESSED SAMPLE SOLUTIONS for all or a representative subset of biological test samples	A small aliquot of the processed sample solution from all of the biological test samples are pooled together and sub-aliquots of this pooled sample are placed in autosampler vials/96-well plates for analysis	Cellular and tissue-based samples	(1) Most representative pooled sample for cellular and tissue samples; (2) represents variation introduced during data acquisition and raw data processing, variation associated with sample processing is not assessed
5	An ARTIFICAL QC SAMPLE created with authentic chemical standards and a dummy sample matrix	Authentic chemical standards for as many metabolites as is achievable or representing as many metabolite classes as is possible are dissolved in an artificial sample matrix (e.g. saline)	Biofluids including tears and breath samples	(1) Provides a measure of data quality but at its lowest representative level; (2) not all metabolites present in the biological test samples will be present in this pooled QC sample; (3) the biological matrix is not accurately represented

The preparation of pooled QC samples is dependent on the type of sample to be studied and the volume of each sample available. The authors recommend, whenever possible, option 1 followed by option 2 in Table 1. Option 3 should only be used as a last resort, or when test samples are only available at low volumes. In this instance, the choice of the alternative biological source is important, as its metabolite composition will determine the metabolites reported in the QC assessment. These approaches can be applied for common sample types such as culture media or mammalian biofluids, but e.g., some biofluids can only be collected in extremely small volumes (e.g. human tears). Where alternative sources are not readily available or may prove to be too expensive to be practical, the preparation of an artificial QC sample (option 5) may be considered but significant care should be taken over the interpretation and value of the resulting QC data.

For cellular and tissue samples, different options are available. The preparation of a single pooled and homogenous sample fully representative of the biological test samples is not possible prior to sample extraction. However, there are two options for preparing a reasonable substitute. The first is to combine small aliquots of each of the extracted samples prior to analysis; this can be before any extract drying process or following reconstitution of dried extracted samples. Data collected for these samples represents variation associated with data acquisition and data pre-processing to convert raw data files in to a data matrix but not sample processing, and this should be clearly defined when quality is reported. The capability to do this is dependent on the mass of tissue, and/or volume of extracted sample, as well as the number of pooled QC samples to be prepared. For tissue samples, excess tissue from the same subjects could be collected if ethically appropriate and applied to separately generate a pooled QC sample. If these options are not possible then the same sample type should be applied from a different biological source of the same species or if not available a representative different species. The second option, specific for cellular samples, is to culture the same cell line in parallel, extract these surrogate “QC” samples, and pool the extraction volumes. Again, this allows the variation associated with data acquisition and pre-processing to be performed but does not include variation associated with the sample preparation for the study samples themselves.

Breath analysis (breathomics) is used for diagnostics of diseases of the lung (Rattray et al. 2014; Lawal et al. 2017). By its very nature breath contains volatile organic compounds (VOCs) that need to be captured before mass spectrometry. As such, it is not feasible to prepare a single pooled and homogenous VOC sample, due to their lability, volatile nature, and the way in which they are captured and pre-concentrated. Compared to more common biofluids, the implementation of QCs for VOC samples is to date relatively underdeveloped (Ahmed et al. 2017). What is currently analysed are QA samples, which are used to try and draw some standardization (Herbig and Beauchamp 2014). The QA samples (not referred to as QCs) are reference mixtures of VOCs which are commonly found in breath. There is a recent working group publication discussing the standardization of the process from exhaled condensates of breath (ECB) or trapped VOCs (as well as for nitric acid) through to analysis and the interested reader is directed here (Horváth et al. 2017).

It is important to note that the pooled QC samples and the biological test samples must be processed in an identical way to ensure that the resulting measure of precision is applicable to the biological test samples and provides quality control for the complete metabolomics pipeline. Also, to ensure that QC injections are representative of biological test sample injections, we strongly recommend having separate QC samples in separate vials and injecting from each vial on a single occasion, or small number of injections (maximum of three injections from a single vial) close together in time. This is especially important if the sample/extract contains a large amount of organic solvent, where evaporation from the vial will result in a change of concentration of metabolites in the QC sample changing over time.

## Measurement of precision and detection

In analytical chemistry, the measurement of any analyte present in a given sample is never perfect. There is always some level of measurement error. Measurement error can be broken down into two components: *systematic (determinate) error*, and *random (indeterminate) error* (Philip et al. 1992).

*Systematic error* is a consistent, repeatable inaccuracy, generally caused by an impaired analytical method, instrument, or analyst. Multiple measurements of samples under the influence of a constant systematic error will always converge toward a mean value that is different to the true value. Such measurements are considered “biased”. In untargeted metabolomics, systematic error can be estimated through multiple measurements, but it cannot be known with certainty because, without an internal standard, the true value also cannot be known.

*Random error* has no pattern and is unavoidable in measurement systems. It is an error caused by factors that vary from one measurement to another seemingly without any known reason. In a metabolomics analytical workflow there are many sources of random error. They can be reduced, but not removed, through good design of experiments, together with optimization of analytical methods, instrumentation, and data processing. The aim of the analytical chemist is to reduce the random error in quantification to the point that it is negligible in comparison to the biological variance.

According to ISO 5725 (ISO5725 1994), *precision* refers to “the closeness of agreement between test results … attributed to unavoidable random errors inherent in every measurement procedure”. Within the context of this paper, we will use the term “precision” to refer specifically to “repeatability precision”, which is defined by ISO 5725 as “a measure of dispersion of the distribution of [independent] test results”, where, “independent test results are obtained with the same method on identical test items in the same laboratory by the same operator using the same equipment within short intervals of time”. Typically, the random error measured can be described by a Gaussian distribution, and therefore can be described statistically by calculating the standard deviation of the repeated measurements. In order for this metric to be a useful comparison tool across multiple analytes it is common practice to standardise this measure of dispersion by dividing it by the mean value. So, for the pooled QC sample measurements detected for *metabolite*_*i*_ (vector ${{\varvec{m}}_{i,qc}}$) the relative standard deviation is calculated using Eq. (1), where ${s_{i,qc}}$ is the sample standard deviation, and ${\bar {m}_{i,qc}}$ is the sample mean.

1 $$RS{D_{i,qc}}=\frac{{{s_{i,qc}}}}{{{{\bar {m}}_{i,qc}}}} \times 100\%$$

In untargeted metabolomics, the relationship between measured analyte (peak area) and actual concentration is often nonlinear and thus a Gaussian error in actual metabolite concentration will not translate to a Gaussian error in measured value. In which case, it may be preferable to calculate the nonparametric statistical equivalent to standard deviation, median absolute deviation (*MAD*). *MAD* can be used as an unbiased estimate of the standard deviation by multiplying by the scaling factor 1.4826 [Hoaglin et al. 1983]. From this, we can derive the robust estimate of relative standard deviation, $RSD_{i}^{*}$ described by Eq. (2).

2 $$RSD_{{i,qc}}^{*}=\frac{{1.4826 \times MA{D_{i,qc}}}}{{median({{\varvec{m}}_{i,qc}})}} \times 100\%$$

An alternative standardised metric for describing the measurement precision of a detected metabolite can be calculated by focusing on the statistical dispersion (i.e. variability, scatter, or spread) of the pooled QC samples in relation to the dispersion of the biological test samples, rather than the average metabolite concentration, as practically demonstrated in several papers (Dunn et al. 2011b; Lewis et al. 2016; Reinke et al. 2017). In this paper we define a similar metric called the “Dispersion ratio” (D-ratio). If the distribution of both the biological test sample measurements and the QC random error are Gaussian then the *D-ratio* for *metabolite*_*i*_ can be defined by Eq. (3), where ${s_{i,qc}}$ is the sample standard deviation for the pooled QC samples, and ${s_{i,sample}}$ is the sample standard deviation for the biological test samples. Again, if the distribution of data is not Gaussian then, the raw data either needs to be mathematically transformed, e.g. log_10_, before calculating the D-ratio, or a non-parametric alternative used, for example see Eq. (4).

3 $$D{\text-}ratio_{i} = \frac{{s_{{i,qc}} }}{{s_{{i,sample}} }} \times 100\%$$

4 $$D{\text-}ratio_{i}^{*}=~\frac{{MA{D_{i,qc}}}}{{MA{D_{i,sample}}}} \times 100\% ~$$

Untargeted metabolomics assays consist of multiple procedural steps in a linear workflow. Before interpreting the D-ratio it is important to consider how the different random error characteristics for each step combine to produce the overall workflow error. Random errors can either be additive or multiplicative, depending on the measurement transfer characteristic of each step of the workflow. The total random error can be expected to be almost exclusively either additive or multiplicative, because the additive effect of two random errors is such that only the larger error significantly impacts the final measurement if it is more than double the other error (Werner et al. 1978). If we assume that the overall measurement error in untargeted metabolomics is dominated by an additive random error structure, then the total random variance measured can be simplified to: $\sigma _{{i,total}}^{2}=\sigma _{{i,biological}}^{2}+\sigma _{{i,technical}}^{2}$, where ${\sigma _{i,biological}}$ is the unobserved biological variance for metabolite_*i*_, and ${\sigma _{i,technical}}$ is the sum of all the unwanted variances accumulated while performing all of the processes in the workflow. We now assume that sample standard deviation of the pooled QCs, $s_{{i,qc}}^{2}$, is a good approximation of complete technical variance, and the sample standard deviation of the biological test samples, $s_{{i,sample}}^{2}$, is a good approximation of the total variance. So, Eq. (4) can now be approximated as Eq. (5), where the denominator describes the Euclidean length of the ${\sigma _{total}}$ directional vector, given that $\sigma _{{biological}}^{2}$ is orthogonal to $\sigma _{{technical}}^{2}$.

5 $$D{\text-}rati{o_i} \approx \frac{{{\sigma _{i,technical}}}}{{\sqrt {\sigma _{{i,biological}}^{2}+\sigma _{{i,technical}}^{2}} }} \times 100\%$$

From Eq. (5), a *D-ratio* of 0% means that the technical variance is zero, i.e. a perfect measurement, and all observed variance can be attributed to a biological cause. A *D-ratio* of 100% indicates that the biological variance equals zero, and the measurement can be considered as 100% noise with no biological information. So, when assessing a given *metabolite*_*i*_, the closer the D-ratio is to zero the better, with the aim of $\sigma _{{biological}}^{2} \gg \sigma _{{technical}}^{2}$.

A descriptive statistic complementary to the estimate of precision is the detection rate. This can be defined as simply the number of detected QC samples divided by the number of expected QC samples for a given metabolite, expressed as a percentage. The detection rate provides a very simple measure of whether a metabolite is consistently detected across a given study. If the detection rate is low, then the reliability of any subsequent statistical analysis on that metabolite will also be low.

These three calculations (*RSD, D-ratio*, and *detection rate*) provide a measurement of quality that can be reported for each detected metabolite and can also be used to remove low quality data from the dataset prior to further univariate or multivariate analysis. In this process of “data cleaning”, acceptance criteria for each metric are predefined and then applied to each metabolite in turn, removing metabolites where the acceptance criteria have not been fulfilled. The acceptance criterion for detection rate is typically set to > 70%, the acceptance criterion for RSD is typically set to < 20% (Sangster et al. 2006; Dunn et al. 2011b) or < 30% (Lewis et al. 2016; Want et al. 2010) depending on the sample type, and it is recommended that the acceptance criterion for *D-ratio* is set to, at most, < 50% (preferably much lower). This process, when combined with removal of blank-related metabolites, can often result in up to 40% of metabolites being removed from the dataset. Although this can be a significant volume of data, the confidence the investigator may place on the remaining data is much higher.

**Fig. 3:**
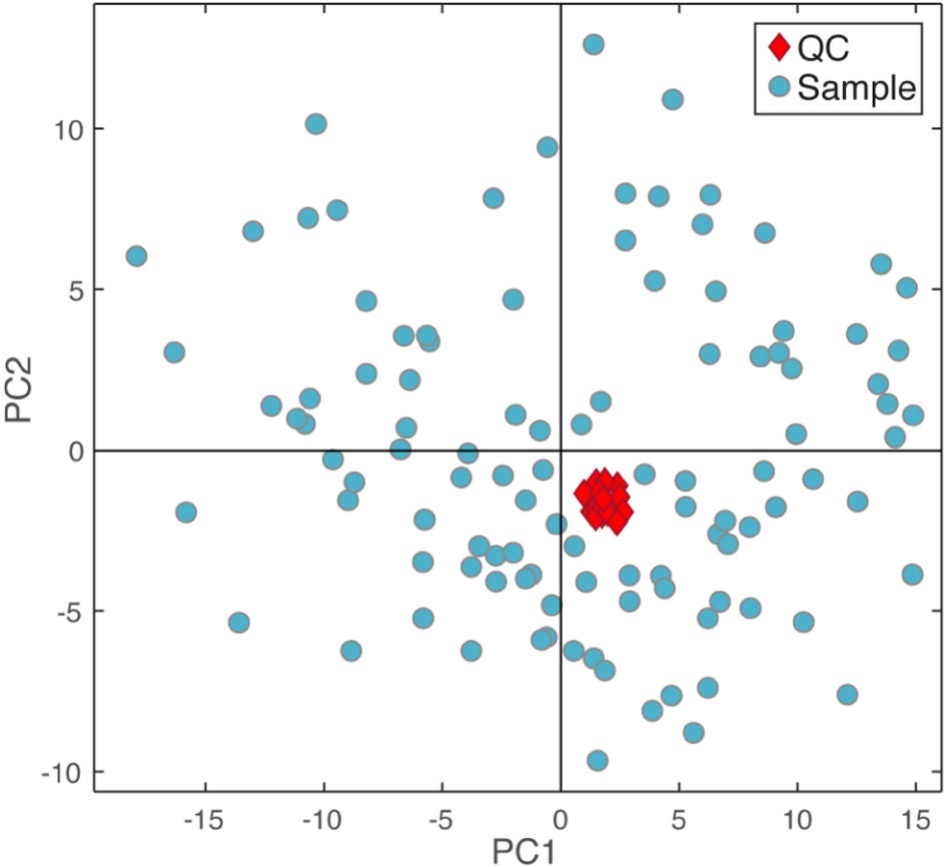
A typical PCA scores plot for a data set deemed of high quality, as the QC data points cluster tightly in comparison to the total variance in the projection

A rapid systematic check of data quality before or after data cleaning can be made by performing principal components analysis (PCA) on the complete data set (suitably scaled and normalized). Then by plotting the first two principal components scores (a projection describing the maximum orthogonal variance in the data) and labelling the data points as either QC samples or biological test samples, the difference in multivariate dispersion can be visually assessed. Figure 3 shows a typical PCA plots for a data set deemed of high quality. Here, one observes that the QC data points cluster tightly in comparison to the total variance in the projection. Ideally the QCs should cluster at the origin of the PCA scores plot, as prior to PCA implementation the input data are mean centred. Any deviation from the origin is usually due to unavoidable pipetting errors or sample weight discrepancies, or when the pooled QC is not generated from sub-aliquots of all the biological test samples. As long as the QCs cluster tightly, relative to the observed dispersion of biological samples, then these data can be deemed as of high quality.

It cannot be emphasised enough that the primary reason for including multiple pooled QC samples in any given study is to calculate a measure of precision for each metabolite detected in that pooled QC sample. These calculations can be made for a single batch or be extended to include as many batches as there are pooled QC samples drawn from a single homogeneous source. The result is a measure of within-batch precision, between-batch precision, and total precision for pooled QC samples drawn from a single source.

## Analytical platform conditioning

In addition to measurement of precision, there are three further uses of the pooled QC sample in untargeted metabolomics studies. Firstly, pooled QC samples can be used to equilibrate, or “condition”, the analytical platform prior to running an analytical batch. This allows for matrix coating of active sites (which can absorb metabolites) in the analytical system, whilst allowing both the chromatography platform, and mass spectrometer to equilibrate. This conditioning process allows higher reproducibility data to be attained for the biological test samples by removing variability in retention times and stabilizing detector response (Zelena et al. 2009). This is not a QC process directly; it is applied to increase data quality.

There is considerable debate on the exact number of conditioning injections required, as this is dependent on multiple factors including the type of sample under analysis, the chromatography system, the injection volume, the chromatography column applied and the mass spectrometer design. Each laboratory should determine the optimal number of conditioning QC injections for each analytical platform and sample type through the injection of 50 pooled QC samples and defining the number of injections where stable data starts to be acquired. Also, using larger sample volumes for the conditioning, may reduce the number of injections required (Michopoulos et al. 2009). We recommend that each laboratory determine the optimal number of conditioning samples for their particular platform, bearing in mind that this number will be different for different operating conditions and sample types. Importantly, the data acquired during this conditioning phase will be more variable than will be observed once the system is conditioned and so it is essential that the data from the conditioning samples are removed from any subsequent data processing, including when calculating each metabolites precision.

## Systematic measurement bias

**Fig. 4:**
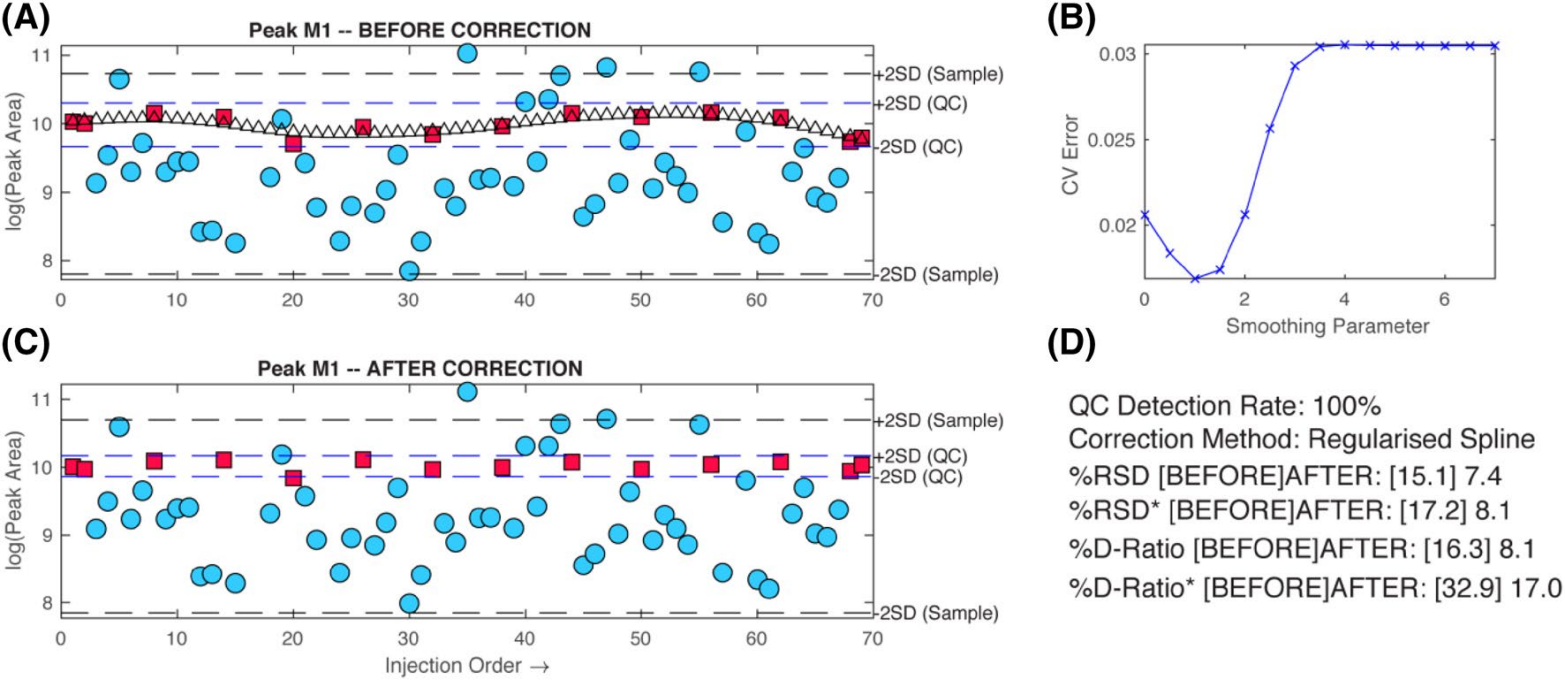
For a given metabolite peak, the measured response can be plotted against injection order (excluding conditioning samples and blanks) and the time varying systematic variation in metabolite response observed (**a**). The systematic variation can be modelled, in this case using a regularised cubic spline with a smoothing parameter. The optimal smoothing parameter value is the one with the lowest cross-validated error (**b**). The ‘correction curve’ can then be subtracted from the raw data (**c**). Accurate measures of precision after the correction can then be calculated (**d**). Red squares are QC samples, blue circles are study samples

The second further use of the pooled QC sample data is for modelling and correcting for systematic measurement bias. If the measured response for a given metabolite is plotted against injection order (excluding conditioning samples and blanks), time related systematic variation in the reported metabolite response can often be observed (see Fig. 4). This systematic error can result from non-enzymatic metabolite conversion (e.g. oxidation or hydrolysis) of samples in the autosampler or from changes in the properties of the analytical platform caused by changes in chromatography (retention time or peak shape) or interaction of the sample components with the surfaces of the chromatography system and MS instrumentation (e.g. column, cones, ion skimmers, transfer capillaries) and therefore influencing measured response (Sangster et al. 2006; Dunn et al. 2011b; Lewis et al. 2016). These effects are dependent on the type of chromatography, analytical system, type of sample, and number of sample processing steps applied.

Systematic error is observed in almost all untargeted data sets. The direction of change and degree of nonlinearity is dependent on the metabolite. Some metabolites show minimal drift, some metabolites show a significant linear increase in response over time, some a linear decrease over time, and many show a nonlinear change over time. If possible, it is advantageous to correct each metabolite mathematically for this systematic error. Doing so will not only allow for a more accurate measure of precision, it will also remove a source of variance which may confound subsequent statistical analysis. The relationship between time, $t$, and response vector, $~{{\varvec{m}}_i}$, for a given metabolite_i_ can be described by Eq. (6), where ${m_{i,j}}$ is the measured response for metabolite *i*, at time-point *j*, ${f_i}(t)$ the time dependent systematic error function, ${\bar {m}_i}$ is the mean response for metabolite *i*, and ${\varepsilon _i}$ is a random variable describing the distribution of the test samples around the systematic error function, We will assume that ${\varepsilon _{i,total}}$ has a Gaussian distribution with variance, $\sigma _{{i,total}}^{2}$

6 $${m_{i,j}}\,=\,{\bar {m}_i}+{f_i}\left( {{t_j}} \right)+{\varepsilon _{i,total}}$$

Now, if we assume that the pooled QC samples are sufficiently representative to describe both the random and systematic technical error for metabolite *i*, then we can also assume that Eq. (7) is true, i.e. ${f_i}(t)$ is the systematic error function for both the test data and the pooled QC data. We will also assume that ${\varepsilon _{i,qc}}={\varepsilon _{i,total}}$ (i.e. ${\varepsilon _{i,qc}}$ has a Gaussian distribution with variance, $\sigma _{{i,total}}^{2}$).

7 $$m_{{i,j_{{qc}} }} {\mkern 1mu} = {\mkern 1mu} \bar{m}_{{i_{{qc}} }} + f_{i} \left( {t_{{j_{{qc}} }} } \right) + \varepsilon _{{i,total}}$$

If all these assumptions hold, then ${f_i}(t)$ can be estimated using any linear or non-linear function that is optimised by the least squares method using the pooled QC sample data, and then the corrected data, ${z_{i,j}}$ can be calculated by the simple subtraction described by Eq. (8) 

8 $$z_{{i,j}} = \varepsilon _{{i,total}} + \bar{m}_{{i_{{qc}} }} = m_{{i,j}} - f_{i} \left( {t_{j} } \right)$$

Many algorithms have been developed to approximate the true systematic error function. Methods include linear regression (van der Kloet et al. 2009), bracketed local linear regression (van der Kloet et al. 2009), LOESS regression (Dunn et al. 2011b), regularized cubic spline regression (see Fig. 4) (Kirwan et al. 2013), support vector regression (Kuligowski et al. 2015), and cluster-based regression (Brunius et al. 2016). All have their theoretical advantages and disadvantages, and no single method is clearly superior; however, there has been a tendency for some of these methods to be implemented without due care by third parties. Most of the methods require the optimization of at least one “smoothing parameter”. The value of this parameter determines the degree in which the regression curve fits to the non-linearity in the data. There is a danger that if this parameter is not sufficiently constrained then the regression curve will begin to fit to the random error in addition to the systematic error in the data. This will negatively impact on the quality, and usability, of the recovered data, but counterintuitively the reported precision will be unrealistically good. This is clearly very dangerous, as the unsuspecting scientist will read the precision report and assume that the data are better than they actually are. As such, it is critical that some form of validation is performed during the model fitting process. One approach is to create a random hold-out set of pooled QC samples (approximately 1/3rd). The correction function is optimised using 2/3rds of the pooled QC samples (training set) and the hold-out set (test set) is used to report the precision measurement (van der Kloet et al. 2009). This method is effective but wasteful of precious QC data, which may result in a significantly inferior generalised model. A more efficient approach is to use, for example, *k*-fold or leave-one-out cross validation (Kirwan et al. 2013; Wen et al. 2017). These methods are standard practice for model optimization in the machine learning community, and cleverly use all of the data in both the training and testing of the regression curve. It has been shown that, after QC based correction with cross validation, the precision of the QC random error is similar to the precision of technical replicates of biological test samples blinded to the modelling process (Kirwan et al. 2013; Ranjbar et al. 2012). As an aside, it is also worth noting that there have been several attempts to correct for systematic error without using pooled QC samples (Rusilowicz et al. 2016; Wehrens et al. 2016); however, these methods are not recommended by the authors.

**Fig. 5:**
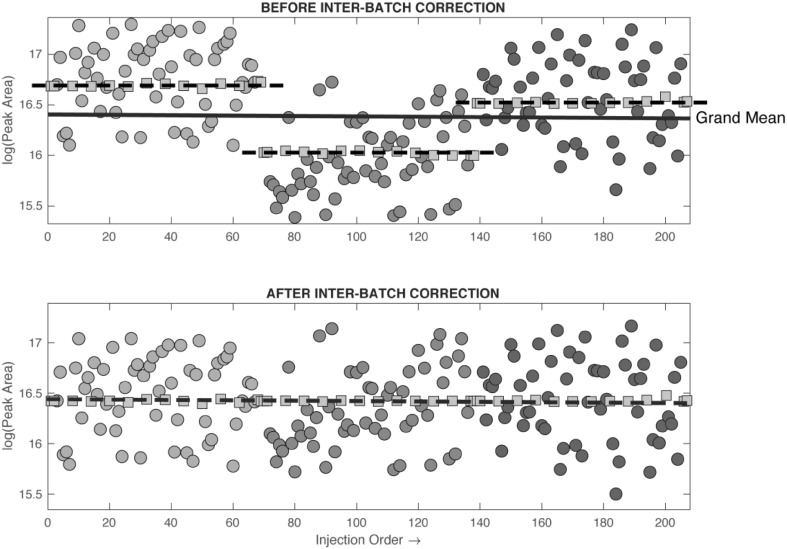
When pooled QC samples drawn from an identical source are used across multiple analytical batches then it is also possible to correct for inter-batch systematic error. First, a grand mean is calculated across all batches, and then difference between each batch mean and the grand mean is subtracted from all the samples in that batch. Red squares are QC samples, blue, green and yellow circles are study samples from batches 1, 2, and 3, respectively

If pooled QC samples drawn from a single homogeneous source are used across multiple analytical batches, then it is also possible to correct for between-batch systematic error. Often, step changes in sensitivity can be observed between batches. Once within-batch systematic error has been corrected then multiple batches can simply be aligned by mean response. Typically, a grand mean is calculated across all batches, and then error between each batch mean and the grand mean is subtracted from all the samples in that batch (see Fig. 5). This process has previously been described in detail (Kirwan et al. 2013).

Real-time correction for instrument sensitivity has also been reported, where the detector voltage is rapidly calibrated after each analysed sample to maintain a consistent measured response across the analytical batch. This has been applied for one thousand urine samples in a single analytical batch producing high precision data with minimal need for post-acquisition correction (Lewis et al. 2016).

## Other QC sample types

The use of pooled QC samples can be expanded further by way of a pooled QC serial dilution. Here, a set of pooled QC samples are diluted in a defined range (e.g. dilution factor range of 1–100%) and analysed to ensure a positive correlation between observed signal and metabolite concentration, satisfying this inherent assumption made in data interpretation (Lewis et al. 2016). The use of linear correlation as a metric of quality clearly biases the data cleaning process toward those metabolite peaks that respond linearly within the range of sample dilution. It does take into account severe non-linearity, and it does not take into consideration the measured response of any biological sample concentrations appearing above the undiluted pooled QC concentration. It is also worth mentioning that calculating the linear correlation coefficient does not provide a metric for peak sensitivity (i.e. the angle of slope between observed signal and metabolite concentration). Also, when interpreting this data, it must be held in mind that dilution of the entire matrix may not fairly represent dilution of any given metabolite within an otherwise static matrix due to idiosyncrasies particularly evident in electrospray ionisation (e.g. ionisation suppression and enhancement). Including a concentration process for the QC sample (e.g. by drying and solubilisation in a smaller volume of sample diluent) for this purpose is not advised due to the potential for broad changes in the matrix. Further work is required to extend the utility of this approach.

### Process internal standards

An approach for providing rapid assessment of data quality for each test sample, independent of any pooled QC sample is to add to each test sample a mixture of multiple compounds (internal standards) of predetermined concentrations representative of the metabolite classes in the test sample metabolome. It is then a relatively straightforward computational process to measure the *m*/*z*, retention time, chromatographic peak shape, and peak area, for each internal standard, for every biological test sample. These data can then either be compared to a theoretical expected value, and criteria for warning of systematic failure during the analytical run can be defined (such as a given parameter moving outside of a specified tolerance interval) or be used to simply monitor systematic changes in parameter values over time (parameter drift), thus providing indication, at a systems level, for the likely need for computational adjustment, after data acquisition, but before statistical analysis or need for instrument maintenance.

One advantage of this internal standard methodology over the pooled QC approach is that data collection and assessment (*m*/*z*, retention time, peak area, peak shape) for each sample can be performed independently of any other sample in the batch. Remember, for untargeted metabolomics it is a computational requirement that raw spectra be deconvolved into a metabolite table after ALL data has been collected as untargeted peak filtering, alignment, and grouping require a consensus algorithm. As such, accurate QC cannot be performed until the end of a batch, or complete study, and only after the completion of this time-consuming deconvolution process. So, it can be considered a *post-data acquisition* quality control process. Conversely, the internal standard QC monitoring can be performed manually for any sample immediately after the raw data are acquired, or potentially implemented as an online real-time monitoring system. In this way, analytical runs can be stopped mid-batch directly after a catastrophic event and system checks/cleaning/restart performed (saving valuable test samples), or “failed” individual samples may be re-injected at the end of the same batch, without too much disruption to the overall workflow. However, it is important to note that the results determined from a small number of compounds do not comprehensively show that all the data generated are of suitable quality. They do, however, provide a simple measure of performance of the analytical platform online during an analytical batch.

The internal standards are typically isotopically-labelled metabolites, although non-isotopically labelled metabolites that are guaranteed to not be present in the biological test samples can also be applied. The authors recommend the use of isotopically-labelled metabolites. The choice of which internal standards to apply is dependent on commercial availability, cost, and physicochemical properties. As the solution will be spiked into all samples, inexpensive internal standards are preferred because of the mass of each internal standard required for each biological study. The internal standards should also provide a broad coverage of physicochemical properties, for the analytical platform applied this should cover a wide range of *m*/*z* values and retention times and therefore include different classes of metabolites with different chemical functional groups. The choice of internal standards will also be based on the analytical method applied, the choice for a hydrophilic interaction liquid chromatography (HILIC) assay will typically be different than that for a lipidomics method. This is because the polar metabolites that are retained on a HILIC column typically elute early in a lipidomics assay and therefore do not meet the criteria of a broad range of retention times. A number of groups have published recommendations (for example, Dunn et al. 2011b; Lewis et al. 2016; Soltow et al. 2013).

The step in the sample preparation process at which the internal standards are added defines the steps for which any variation is measured. When the internal standards are added at the final preparation step and after sample processing, then variation associated with data acquisition only can be recorded. However, if the internal standards are added to the biological samples before they are processed then variation associated with sample processing and data acquisition processes are recorded, albeit only for those compounds used as internal standards. One option is to add some internal standards before sample processing and some internal standards before sample analysis to allow both options to be applied. The choice is for the analyst though reporting of data quality should define when the internal standards were added.

It is important to note that this mixture of internal standards is used to ONLY monitor the performance of the system and should NOT be confused with the internal standards used in quantitative analysis for determining analyte concentrations. Their use for quantification has been suggested by some groups. However, for untargeted studies using a small selection of internal standards to act as quantifiers for all detected metabolites is extremely difficult, if not impossible. This is because the chemical identity of all metabolites, and their associated calibration curves, is typically not known before or after data acquisition. In addition, ionization is structure dependent, and can vary significantly across a class, and that ion suppression is usually retention time dependent.

### Standard reference materials (SRMs) and long-term reference (LTR) QC samples for inter-study and inter-laboratory assessment of data

While all of the above QC samples allow assessment of data quality within a single laboratory and single study, they do not allow data quality comparisons across different studies within a laboratory or across different laboratories. To address this concern, standard reference materials or a different type of pooled QC sample can be applied. For intra-laboratory and inter-study data quality assessment, a laboratory pooled QC sample can be prepared by purchasing the sample type in large volumes from a vendor or preparing it from a range of individuals from within the study facility and thoroughly mixing these samples together to create a single pooled QC sample (Dunn et al. 2011b; Begley et al. 2009). This sample can then be sub-aliquoted and stored at − 80 °C or in liquid nitrogen. One or multiple aliquots can then be processed and analysed for each analytical batch and/or multi-batch study. The data acquired provides a long-term assessment of data within the laboratory. The use of this type of sample (also called a LTR) does not preclude the use of a pooled QC obtained from the study biological samples in the same run as these QCs perform slightly different functions. The LTR allows data from different batches to be compared, whilst the data from the study-derived samples will potentially provide a more relevant QC for that particular batch (see, Lewis et al. 2016).

A standard reference material provides a method to allow quality assessment across different laboratories. SRMs are created and sold by a certified group, with NIST providing the most widely applied. The SRM can be purchased by different laboratories and the data reported from each laboratory. Currently, SRM1950 (Simon-Manso et al. 2013) is the most widely used plasma SRM in metabolomics as shown by a number of inter-laboratory comparison studies (Bowden et al. 2017; Siskos et al. 2016). SRM’s may be considered an expensive option for routine use; however, they have the advantage over LTR with respect to considerations for long-term stability of pooled QC storage at − 80 °C, if liquid nitrogen storage is not an option. Sample stability is an important long-term QA process and a small number of papers have reported stability of metabolites (for example see, Haid et al. (2018) for a human plasma study).

## The order of QC samples in analytical batches

**Fig. 6:**
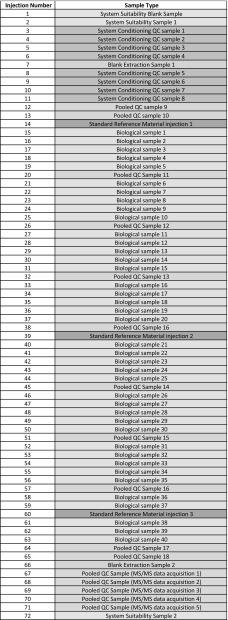
A typical analysis order applied for an untargeted metabolomics assay is composed of system suitability samples at the start and end of the analytical batch and pooled QC samples analysed at the start of the run (typically 10 injections with 8 system conditioning QC samples followed by 2 QC samples for QC processes and signal correction), at the end of the run (typically 2 injections) and periodically during the analysis of biological samples (typically every 5–10 biological samples). A system suitability blank sample is analysed at the start of the analytical batch, a blank extraction sample is typically analysed twice, and a standard reference material is analysed three times during an analytical run. If MS/MS data acquisition is not applied for each biological sample, then a set of pooled QC samples can be applied separately at the end of the run for MS/MS data acquisition

In Sect. 2 we abstractly discussed the effectiveness of five different types of QC samples in a given analytical batch. However, the true effectiveness of these samples is heavily dependent on how many times each type of QC sample is analysed, and at which positions in the run should they be placed. Figure 6 shows an example of a routinely used, and effective, untargeted analytical run order following routine maintenance, cleaning of the analytical platform, and successful application of system suitability tests. Here, eight pooled QC samples are analysed at the start of each batch to condition the platform, and their data removed prior to data processing. Pooled QC samples are then analysed periodically throughout the batch. In this example pooled QCs are analysed every 5th sample; however, the required frequency is dependent on several independent factors each potentially contributing to varying degrees of systematic non-linear change in peak-area sensitivity, individual to each detected metabolite. These factors include: complexity of sample matrix, specific instrument dynamics, batch length, and data processing software. As such, it is recommended that each laboratory develop their own “fit for purpose” process. However, for accurate QC assessment, it is recommended that a pooled QC is analysed at least every 10th sample, and/or there are at least five pooled QCs distributed evenly across a single batch. If a nonlinear signal correction algorithm is to be used, then it is recommended that at least eight pooled QCs samples are used. Additionally, as a safeguard against possible QC miss-injection, it is also recommended that two QC samples are analysed at the beginning and end of the batch, before and after all test samples have been run (in this example, pooled QC sample pairs 9/10, and 17/18). The first and last pooled QC samples are disproportionally influential in constructing the signal correcting regression curves, and if missing the resulting models will be forced to extrapolate rather than interpolate with unpredictable results. If leave-one-out cross-validation is being used to optimise the signal correcting regression curves, then it may be preferable to include three, rather than two, pooled QC samples at the beginning and end of each batch. This will act as a further safeguard against extrapolation during optimisation; however, it is more efficient to implement leave-one-out cross-validation such that the end QCs are never left-out.

Two types of “blank” sample can be analysed in an analytical batch. The first type of blank sample is analysed at the start of each batch and is part of the system suitability tests, not the QC process. Here, an analysis is performed with no injection, or an analysis is performed with injection of a contaminant-free solvent. We will define this as a **system suitability blank** sample. The second type of “blank” sample is a **process blank** sample. Here, analysis is performed on a sample that has been prepared in a manner identical to that of the biological samples except that the actual biological sample (serum, urine etc.) is replaced with a solution. This process blank (also commonly known as an extraction blank) provides information on the detection of peaks related to (a) contaminants included during sample preparation, which are not metabolite peaks and (b) sample carryover usually due to inadequate washing of the LC injection system between sample injections. We recommend that three blank samples are analysed in each batch. The system suitability blank is the first injection of any analytical batch. The process blanks are injected midway through column conditioning to cleanly measure “systematic” contamination, and the second at the end of a batch immediately after the final pooled QC, to measure cumulative “carryover” contamination. It is important to note that position of the process blank in the injection order must be decided such that no test sample directly follows a blank QC because a single blank extraction will disturb the equilibrium of the platform, significantly de-condition the column, and adversely affect the quality of the data for samples analysed immediately after. It is important to note that typically after a blank injection 4 or 5 pooled QC samples need to be injected to re-condition the system before another biological sample can be accurately analysed; again, the exact number should be determined by each laboratory.

The intra-study or intra-laboratory pooled LTR QC or SRM is typically analysed up to three times in a single study. This allows variation across a batch to be monitored as well as variation between batches and studies to be monitored. Finally, if MS/MS data are not collected for all of the biological samples then a set of pooled QC samples can be applied at the start or the end of the analytical batch for MS/MS data acquisition and can be used to support metabolite annotation [see Mullard et al. (2015) for a discussion on applying different data dependent acquisition (DDA) experiments for each injected pooled QC sample].

## Summary

The application of untargeted metabolomics to biomedical and clinical research is now a global phenomenon, but, the adoption of global standardised workflows for sample processing, data acquisition, and data processing has not yet been achieved. In the current research climate, particularly with such a diverse range of hyphenated platforms produced by many manufacturers, a single unified QA/QC procedure will not fit all laboratories. The guidelines presented here have been primarily written to promote good practice, both in application and reporting. We have discussed different types of system suitability and QC samples that can be used in untargeted MS-based metabolomics. Each protocol is relatively easy to implement, and achievable in both small and large laboratories. We have argued the unique importance, and applicability of each type of system suitability and QC sample; described the metrics that can be used to enable confidence in both the ongoing reliability of a given analytical platform and provided advice on how to ensure the collection of high quality data. The authors highly recommend the use of all the system suitability and QC sample types presented, whether performing a short single-batch analysis, or embarking on a large-scale multi-batch study. As a minimum requirement, we suggest the use of the system suitability samples, blank process sample, and the pooled QC sample. However, every laboratory needs to optimize their methods to best fit their situation.

Currently, within the clinical metabolomics community, there is massive inconsistency in the reporting of data quality in scientific publications, and data repositories. The development of community agreed QA/QC reporting standards is urgently needed. Robust workflows including comprehensive QC reporting will only enhance the reproducibility of results, facilitate the exchange of experimental data, and build credibility within the greater clinical scientific community. Moreover, we strongly endorse that the data generated from these QCs are published along with the study and deposited in suitable metabolomics databases or repositories.
